# Ecological prevalence and genomic characterization of *Salmonella* isolated from selected poultry farms in Jiangxi province, China

**DOI:** 10.1016/j.psj.2025.105197

**Published:** 2025-04-24

**Authors:** Xiaowu Jiang, Abubakar Siddique, Lexin Zhu, Lin Teng, Sajid Umar, Yan Li, Min Yue

**Affiliations:** a College of Medicine, Yichun University, Yichun, Jiangxi, 336000, PR China; b Laboratory of Animal Pathogenic Microbiology, Yichun University, Yichun, Jiangxi, 336000, PR China; c Department of Veterinary Medicine, College of Animal Sciences, Zhejiang University Hangzhou, 310058, PR China; d Key Laboratory of Systems Health Science of Zhejiang Province, School of Life Sciences, Hangzhou Institute for Advanced Study, University of Chinese Academy of Sciences, Hangzhou, 310024, PR China; e Global Health Research Center, Duke Kunshan University, Suzhou, 215316, Jiangsu, PR China; f Zhejiang Provincial Key Laboratory of Preventive Veterinary Medicine, Hangzhou, 310058, PR China

**Keywords:** *Salmonella*, Poultry feces, One Health, Multi drug resistance, Whole genome sequencing, Jiangxi, China

## Abstract

Non-typhoidal *Salmonella* (NTS), particularly antimicrobial-resistant serovars, remains the major source of foodborne bacterial illnesses. Raw chicken is the leading cause of human salmonellosis. In this study, we evaluated the prevalence, antimicrobial resistance profiles, and genomic features of 143/1,800 (7.94%) *Salmonella* strains isolated from poultry farms in five major regions of Jiangxi province, China, between 2022 and 2023 using Whole genome sequencing (WGS). Among *Salmonella* isolates, the most common serovars were Infantis (ST32) and Enteritidis (ST11). Resistance to amoxicillin and tetracycline was the most prevalent, with 60.84% of *Salmonella* isolates exhibiting a multi-drug resistance (MDR) pattern. The detection of antimicrobial-resistant genes (ARGs) examined was aligned with the resistant phenotypes found. A total of 61 ARGs were identified, with *aph(*3*′)-Ia, qnrS1, aph(*3*′')-Ib*, and *tetA* being the prominent ARGs. Furthermore, 24 beta-lactam genes were also identified, including *bla*_TEM_*, bla*_SHV_*, and bla*_CTX-M_. The number of ARGs and the distribution of serovars varied according to the year, farms, and cities. *Salmonella* isolates carried 13 heavy metal resistance genes (HMRGs) and two biocide resistance genes, with *pcoS* being the most prevalent. A total of 145 virulence genes and 19 plasmids were found, with serovars Infantis and Enteritidis having the most virulence genes. The high occurrence of MDR *Salmonella* in this study, particularly carrying numerous mobile genetic elements (MGEs), posed a serious threat to food safety and public health, emphasizing the need to improve poultry farm hygiene to decrease contamination and transmission.

## Introduction

*Salmonella enterica* is one of the most common foodborne pathogens globally (Tang, et al., 2023). Food animals and their derived products (eggs and meat) are potential carriers of these infectious pathogens (Shi, et al., 2021; Tan, et al., 2023; Xu, et al., 2020a). Consumption of these poultry-related products is one of the main global causes of *Salmonella* outbreaks, which poses a serious risk to public (Chen, et al., 2024; Li, et al., 2022b; Siddique, et al., 2021b; Wang, et al., 2024a). *Salmonella*, a gram-negative facultative anaerobic bacterium belonging to the Enterobacteriaceae family, is a foodborne pathogen that infects both humans and animals (Fierer, 2022). Most of the salmonellosis infections are self-limiting, but they can represent a serious threat to life in immunocompromised or elderly patients and may be treatable with appropriate antibiotic therapy (Feng, et al., 2023; Nambiar, et al., 2024; Wei, et al., 2023b; Zhou, et al., 2025). According to the World Health Organization (WHO), 9.87 million incidents of gastroenteritis occur each year, with 91.5% of these cases being due to consumption of contaminated food (Ferrari, et al., 2019). Moreover, *Salmonella* is one of the most common bacterial foodborne pathogens among diarrheal illnesses in China in recent years (Zhou et al., 2025). *Salmonella* is also an important source of antimicrobial resistance (AMR) genes, significantly threatening public health and security (Elbediwi, et al., 2023; Jin, et al., 2024; Tang, et al., 2022).

AMR has emerged as a significant global health concern in the twenty-first century (Odoch, et al., 2017). Resistance to key antibiotics, especially extended-spectrum cephalosporins, carbapenems, and fluoroquinolones, has been reported in both humans and the poultry industry in recent years (Che, et al., 2023). In 2024, the WHO listed fluoroquinolone-resistant NTS as a high-priority pathogen (Beşli, et al., 2024). Poultry products have been linked to the spread of extended-spectrum β-lactamase (ESBL) producing *Salmonella* strains, posing a risk to human health in different countries (Elbediwi, Tang and Yue, 2023). The transmission of extensively drug-resistant (XDR) and multi-drug resistant (MDR) *Salmonella* through ingestion of raw or undercooked poultry products is a substantial food safety as well as public health concern globally (Elsayed, et al., 2024a). However, the widespread use of antibiotics in farm animals has created selective pressure among bacteria, resulting in the emergence of carbapenem and colistin-resistant *Salmonella* (Ke, et al., 2024b; Siddique, et al., 2021a). Numerous investigations have found a direct link between antibiotic use in food animals and the rise of AMR in foodborne bacteria linked to human illness (Hoelzer, et al., 2011; Jiang, et al., 2025; Li, et al., 2025). A major issue is that regularly exposing these animals to low doses of antibiotics contributes to AMR, since many of the medicines employed are the same or substitutes of antibiotic used in human therapeutic practices (Van, et al., 2020).

However, during the past few years, antimicrobial resistance data and bacterial infections in poultry farms have not been adequately disclosed (Elsayed, et al., 2024b). Recent developments in sequencing technologies have contributed to increased widespread use of whole-genome sequencing (WGS) in epidemiological investigation and transmission of antimicrobial resistant pathogens (Gerner-Smidt, et al., 2019). WGS is being utilized globally as a comprehensive tool for food safety and public health, particularly in the investigation of *Salmonella* outbreaks in both humans and animals (Pornsukarom, et al., 2018). This method demonstrates higher resolution compared to conventional sequencing techniques; the prediction of antimicrobial resistance and virulence genes through WGS can improve our understanding of the risk posed by foodborne pathogens to human health (Wang, et al., 2024b).

Jiangxi is a top chicken-producing region in China with an extensive range of domestic chicken breeds. By the end of 2022, Jiangxi Province had 241.674 million chickens in stock, 4.2% more than the previous year (Tan, Li, Yang, Zhang, Tan, Zeng, Wei, Huang, Wu and Li, 2023). However, research on poultry diseases such as *Salmonella* and the relative antibiotic susceptibility of the Jiangxi poultry industry has not been well reported over the past decades (An, et al., 2025; Jia, et al., 2025; Wang, et al., 2025a; Wang, et al., 2025b). A few studies that investigated the incidence of *Salmonella* in chickens without knowing the current antibiotic resistance phenotypic or genotypic trends in Jiangxi province. In this work, we investigated the prevalence, AMR profiles, and genetic characteristics of *Salmonella* isolated from various poultry farms. This study also highlights the potential advantages of WGS technology as a powerful means for promoting evidence-based test security threats for local policymakers and public authorities.

## Materials and methods

### Sample collection

A cross-sectional study was conducted on poultry farms located in five major cities representing the cardinal directions of East (Fuzhou), South (Ganzhou), West (Pingxiang), North (Jiujiang), and the Central (Nanchang) region of Jiangxi province between 2022 and 2023. The poultry faecal samples were collected from randomly selected six broiler chicken farms in the above-mentioned cities, with a medium inventory housing ≤ 5,000 in each farm per year within Jiangxi province. All of the samples in one site were visited only once. The sampling size in each farm was estimated as ≤ 280 by using the formula as described previously (Mama and Alemu, 2016), Sample Size (n)=z^2^pq/d^2^ and the estimated prevalence (p) is referenced to the previous study in China (24.0% from (Zhao, et al., 2017) and 11.9% from (Li, et al., 2013)), A total of 1800 poultry faecal samples with 300 samples per farm were gathered from six distinct poultry farms as documented (Table 1). Poultry faeces were collected with sterile cotton swabs and placed in sterilized bags. Samples were transferred on ice and stored in refrigeration at 4°C until further analysis at the microbiological biosafety laboratory.

**Table 1:** Sample summary and *Salmonella enterica* positive isolates distributed across poultry farms from different regions in Jiangxi Province.

Geographical Regions	Total no. of Samples	No. of Positive isolates	Positive rate (%)
Fuzhou	300	16	5.33
Pingxiang	300	31	10.33
Jiujiang	600	45	7.50
Ganzhou	300	15	5.00
Nanchang	300	36	12.0
Total	1800	143	7.94

### Isolation and identification of *Salmonella*

The *Salmonella* isolates were isolated and identified using the methodology described in a previous study (Qiu, et al., 2021). For pre-enrichment, swab samples were transferred to 10 mL buffered peptone water (BPW) (Oxoid, UK) and incubated aerobically at 36°C for 18 hours. The enrichment broths were then transferred aseptically into 10 mL of Selenite F broth and incubated at 37°C for 24 h. After completion of the selective enrichment procedure, aliquots of the incubated Selenite F broth were streaked onto xylose−lysine−deoxycholate agar (XLD, Oxoid, UK) using a loop, and each plate was incubated at 37°C for 24 h. Pink colonies with or without black centres on XLD were considered as *Salmonella* and streaked separately on fresh XLD agar plates for biochemical analysis. The isolated *Salmonella* strains were further confirmed through biochemical tests such as Urease, Triple Sugar Iron (TSI), Sulphate, motility assays and indole test. Overnight-grown bacterial cultures were streaked on specific media for these respective tests i.e. Triple Sugar Iron Agar (Oxoid, UK), Urease Agar (Oxoid, UK), Sulphate, Indole Motility (SIM) Agar (HIMEDIA, IND) and Simmons Citrate Agar (Oxoid, UK), and incubated at 37°C for 24 hours. The *Salmonella*-positive bacteria were further identified, through PCR targeting the *invA* gene (Bai, et al., 2018).

### Antimicrobial susceptibility assessment

The antimicrobial susceptibility testing was performed using the broth microdilution method according to National Antimicrobial Resistance Monitoring System (NARMS) guidelines (Mohiuddin, et al., 2020). A total of 13 clinically significant antibiotics belonging to eight different classes were used. The antibiotics used for susceptibility assay were amoxicillin-clavulanic acid (AMC), ampicillin (AMP), chloramphenicol (CHL), ciprofloxacin (CIP), nalidixic acid (NAL), sulfamethoxazole/sulfonamides trimethoprim (SUL), kanamycin (KAN), cefoxitin (CX), ceftiofur (CF), gentamicin (GEN), streptomycin (STR), tetracycline (TET), and azithromycin (AZI). The results were interpreted according to Clinical and Laboratory Standards Institute (CLSI) recommendations (CLSI, 2019). There are no CLSI interpretive standards for cefoxitin, streptomycin, ceftiofur, or azithromycin for *Salmonella*, hence, interpretative criteria defined by the NARMS were used for these antibiotics. The control strain was *Escherichia coli* ATCC 25922. *Salmonella* isolates were classified as MDR if they exhibited resistance to at least three or more than three antibiotic classes. Pan-drug resistance (PDR) was described as the ability to resist all antimicrobial agents in all categories.

### Biofilm formation

The ability of each isolate to develop biofilms was assessed using the standard crystal violet assay in 24-well polystyrene microtiter plates, according to a previously described method (Obe, et al., 2018). An overnight culture of each isolate was prepared in 10 mL TSB and subsequently diluted to a final inoculum of 10^7^ CFU/mL. Two millilitres of the inoculum were distributed into six wells, with wells containing only TSB (non-inoculated broth) serving as negative controls for each isolate. The 24-well plate was incubated at 37°C for 48 hours to facilitate biofilm formation. After a 48-hour incubation period, the inoculum was extracted from each well of the plate, and the wells were washed three times with sterile distilled water to eliminate loosely attached cells. The plate underwent air-drying for 15 minutes to fix the cells, followed by the addition of 2 mL of a 1% crystal violet solution (AC447570500, ACROS Organics) to stain the attached cells in both treated and control wells. The plate was then incubated at room temperature for 20 minutes. Subsequently, the dye was eliminated from the wells, which were then washed five times with sterile distilled water to ensure the removal of all dye residue. Plates were allowed to dry at room temperature and then 150 μL of 30% acetic acid was added to each well to resolubilize the stained. The biofilm formation of each strain in the wells was quantified by measuring the optical density (OD_600nm_) using a microplate reader (Bio-rad, USA). The biofilm formation potential of each isolate was evaluated by calculating the final optical density (OD) values, (Final OD600 = average OD isolate – average OD negative control). The biofilm formation capability of each isolate was then analyzed by dividing them into three categories: 1) Weak biofilm producers exhibited final OD_600nm_ values< 0.3, 2) moderate biofilm producers demonstrated final OD600 values ranging from 0.3 to 0.6, and 3) strong biofilm producers showed final OD_600nm_ values > 0.6 (Obe, et al., 2021).

### Genomic DNA extraction and Whole-genome sequencing (WGS)

The extraction of DNA was performed using a semi-automated QIAcube system using the Roche MagNA Pure 24 platform following the manufacturer's guidelines (Qiagen, CA, United States). The purity of extracted DNA was assessed by measuring the A_260_/A_280_ ratio (∼1.8 for pure DNA) using NanoDrop spectrophotometers, in accordance with the manufacturer's instructions. Quantification of the extracted DNA was measured using the Qubit 2.0 fluorometer (Invitrogen, CA, USA). Library preparation was performed with Illumina DNA prep (previously known as FLEX), and sequencing was accompanied by using MiSeq Reagent Kits v3 (600 cycles). During FASTQ generation, automatic adapter trimming was performed on Illumina MiSeq (Ke, et al., 2024a). Raw sequencing data were demultiplexed using Illumina's bcl2fastq tool (version 2.20), and quality was crisscrossed using Quast (version 4.6.3). FastQ files were then uploaded to the Galaxy web platform, where they were assembled using Spades version 3.12.0 with the default settings on the public server at (usegalaxy.org) (Siddique, et al., 2024a).

### Bioinformatic analysis

After the completion of genome assembly, the predication of serotypes was carried out using SISTR v1.0.2 with the default parameters (Jiang, et al., 2019). To determine the sequence types (STs) of the strains, multi-locus sequence typing (MLST v2.0) https://cge.cbs.dtu.dk/services/MLST/) was used. ResFinder v4.1 (https://cge.cbs.dtu.dk/services/ResFinder/), was used to identify antimicrobial resistance genes (ARGs) with default settings. The VFDB database was used for the identification of virulence factors in the *Salmonella* genomes (Jia, Huang, Zhou, Zhou, Wang, Siddique, Kang, Cao, Huang and He, 2025). SPIFinder v2.0 was used to identify pathogenicity islands (PIs). Plasmid Finder v2.1 (https://cge.cbs.dtu.dk/services/PlasmidFinder/) was used to detect plasmid replicons. Additionally, from *Salmonella* genomes, metal and biocide resistance genes were identified by BacMet v2.0 (http://bacmet.biomedicine.gu.se/) (Tang, Elbediwi, Nambiar, Yang, Lin and Yue, 2022).

### Phenotypic and genotypic AMR correlation

Correlations between phenotypic and genotypic data gathered in this investigation were statistically analyzed using a two-by-two table analysis (Wei, et al., 2023a). The presence or absence of ARGs, derived from WGS data, was compared with MIC phenotypic results that reflect the actual resistance profile of the isolate. Intermediate phenotypes were not considered in this analysis. The correlation between phenotypic and genotypic antimicrobial resistance (AMR) was determined by dividing the total number of phenotypically antimicrobial-resistant *Salmonella* isolates by the total number of *Salmonella* isolates possessing the corresponding resistant genes. The specificity, sensitivity, negative predicted value (NPV) and positive predicted value (PPV) were calculated as previously described (Alzahrani, et al., 2023).

### Data analysis

SPSS 20.0 (SPSS, Chicago, United States) was used for data analysis. The Kruskal-Wallis test was conducted to compare samples from more than two groups. The chi-square test was employed to evaluate the statistical significance of the categorical variables. Heatmaps illustrating the clustering of serotypes, AMR genes, virulence genes, and plasmid replicons were generated using ggplot2 package in R software (R v4.4.1).

## Results

### Prevalence of *Salmonella*

**Fig. 1:**
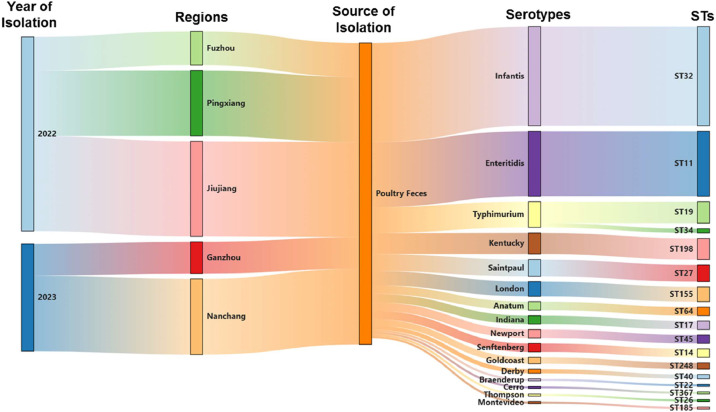
Sankey diagram combining the year of isolation, regions, sampling source and serotypes for 143 *Salmonella* isolates. The diameter of a line is proportional to the number of isolates from the indicated source, which is also shown in parentheses on the right.

A total of 143 *Salmonella* isolates, accounting for 7.94%, were collected from poultry feces in five major cities of Jiangxi Province, China, between 2022 and 2023 (Table 1). These *Salmonella* isolates were collected from different chicken farms in five major cities, including Fuzhou, Pingxiang, Jiujiang, Ganzhou, and Nanchang. The capital city of Nanchang had the highest prevalence of *Salmonella* (12.0%), followed by Pingxiang (10.33%) and Jiujiang (7.5%). These 143 isolates belonged to sixteen serovars based on the in-silico serotyping method. The most common serovar was *S*. Infantis (32.87%, 47/143), followed by Enteritidis (21.68%, 31/143) and Typhimurium (7.69%, 11/143) (Fig. 1). Seventeen distinct ST types were recognized, which corresponded to the sixteen identified serovars. Among the 143 *Salmonella* isolates, ST32 (*S*. Infantis) was the most abundant (32.87%) sequence type, followed by ST11 (*S*. Enteritidis) (21.68%).

### Antimicrobial susceptibility assay

**Fig. 2:**
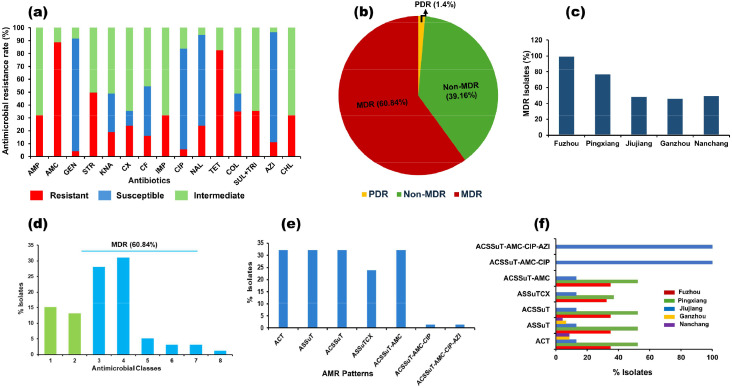
Antimicrobial resistance pattern of 143 strains of *Salmonella enterica* isolated from different regions. a; The prevalence of antimicrobial-resistant isolates for each drug. b; Percentage of *Salmonella* isolates found to be MDR and XDR in our study. c; MDR isolates in each region. d; AMR patterns in different antibiotic classes. e; Different antimicrobial resistance patterns of all 143 *Salmonella* isolates. f; MDR patterns in different regions.

The minimum inhibition concentrations (MICs) were used to define the results of the antimicrobial resistance of *Salmonella* isolates (Supplementary Table S1). The most prevalent resistance phenotypes observed among *Salmonella* isolates were against AMC (88.8%) followed by TET (82.5%). The highest susceptibility was observed against GEN (87.4%) followed by AZI (85.3%) and CIP (78.3%) (Fig. 2a). Multi-drug resistance patterns were reported in (60.84%), while pan-drug resistance patterns were observed in (1.4%). However, according to our results, 39.16% of the isolates were not multi-drug resistant (Fig. 2b). Among *Salmonella* samples, the MDR rate from Fuzhou City was the highest (100%), followed by Pingxiang City (77.42%) and Nanchang city (50%) (Fig. 2c).

### Multi-drug resistance (MDR) patterns

Additionally, we examined several antimicrobial resistance patterns for the isolates, including tri-, tetra-, penta-, hexa- and hepta-drug resistance patterns (Fig. 2d). Among the isolates, more than 32.17% of the isolates were shown as tri (ACT, ampicillin, chloramphenicol, and tetracycline), tetra (ASSuT, i.e., resistance to ampicillin, streptomycin, sulfamethoxazole and tetracycline), penta (ACSSuT, ASSuT and chloramphenicol), or hexa (ACSSuT-AMC, ACSSuT, and amoxicillin-clavulanic acid) MDR patterns. The study also found critical hepta- and octa-MDR patterns of ACSSuT-AMC-CIP (ACSSuT-AMC, ciprofloxacin) and ACSSuT-AMC-CIP-AZI (ACSSuT-AMC, ciprofloxacin and azithromycin) (1.4% each) (Fig. 2e). Interestingly, all the hepta- and octa-MDR isolates have been identified in Jiujiang city isolates (Fig. 2f).

### Resistome profiling of *Salmonella* Isolates

**Fig. 3:**
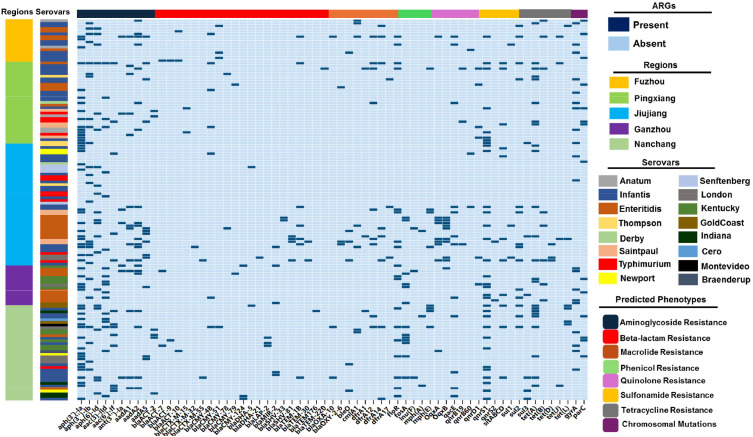
The distribution of antimicrobial resistance genes (ARGs) belongs to different antimicrobial classes among 143 *Salmonella* isolates from poultry farm sources. Genes detected in the genomes associated with AMR are shown on the X-axis of the graph and are grouped by drug classes. The dark blue color in the heatmap represents the presence of ARGs and the light blue color indicates the absence of ARGs.

The WGS sequences of 143 *Salmonella* genomes were analyzed using an in-silico approach to find antibiotic-resistance genes. Sixty-one resistance genes were found to confer resistance to seven different antibiotic classes, which include tetracycline, aminoglycosides, β-lactams, sulfonamides, fosfomycin, quinolones, and macrolides (Fig. 3). The most frequently found ARGs were *aph(3′)-Ia* (37.76%; 54/143) and *aph(3′')-Ib* (17.48%; 25/143) which indicates resistance to aminoglycosides, *qnrS1* (22.38%; 32/143), which encodes resistance to quinolones, and *tetA,* (16.78%; 24/143) which encodes resistance to tetracyclines. The high no. of beta-lactam resistant genes (n=24) has been identified, which confer resistance to narrow spectrum β-lactamases (*bla*_Lap-2_), the extended-spectrum cephalosporins (*bla*_CMY_*_,_ bla*_CTX-M_*_,_ bla*_SHV_ & *bla*_ACT_) β-lactamases; the carbapenem-mediated ESBLs (*bla*_OXA_); and the penicillin-mediated ESBLs (*bla*_TEM-1B_). Additionally, chromosomal mutations in *gyrA* and *parC* have also been identified in the *Salmonella* genomes (Fig. 3). The broad distribution of these important resistance genes in strains derived from poultry is an alarming public health concern.

### AMR correlation based on phenotypic and genotypic data (WGS)

The effectiveness of WGS in predicting phenotypic resistance was evaluated by comparing phenotypic AMR profiles with genome sequence data (Table 2). The phenotypic resistance profiles for tetracycline, macrolides, penicillin, and aminoglycosides revealed the most prevalent antimicrobial resistance phenotypes identified in this study. Phenotypic resistance and the presence of related AMR genes showed a strong overall association using whole-genome sequencing data (Table 2). The highest association between phenotypic and genotypic resistance was observed in tetracycline (90.27%), whereas the lowest was identified in sulphonamides (29.03%). The overall sensitivity of AMR coding genes present for resistance prediction across all antimicrobials was (85.80%), specificity (88.79%), positive predictive value (PPV) (87.85%), and negative predictive value (NPV) (90.29%).

**Table 2:** Comparison between genotypic AMR prediction by WGS and phenotypic expression levels of AMR *Salmonella* isolates.

Antibiotics	Resistant by phenotype		Susceptible by phenotype		Sensitivity (%)	Specificity (%)	PPV (%)	NPV (%)
	WGS: AMR gene +	WGS: AMR gene -	WGS: AMR gene +	WGS: AMR gene -				
Aminoglycosides	102	23	3	15	81.6	84.21	85.46	90.1
Beta-lactams /Cephalosporins	88	33	2	20	72.72	91.30	93.14	86
Quinolones	103	20	3	17	82.73	85.71	80	95.4
Tetracycline	130	13	0	0	90.27	0	80	95.4
Phenicol	115	15	1	12	88.46	85.71	87.16	97.1
Sulphonamides	36	87	3	17	29.03	85.0	91.13	94.6
Macrolides	71	40	6	26	64.2	80.11	89.09	77.9
Total					85.80	88.79	87.85	90.29

+: Presence, -: Absence, PPV: Positive predictive value, NPV: Negative predictive value.

### Biofilm formation and detection of heavy metal and biocide resistant genes

**Fig. 4:**
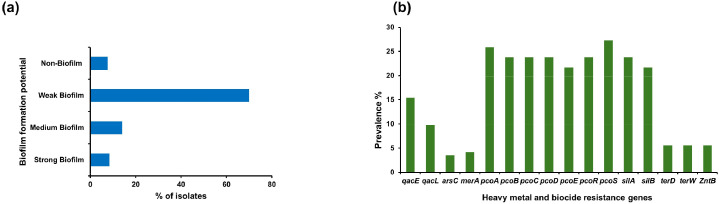
Characteristics of 143 *Salmonella* Isolates. a; Biofilm formation potential of *Salmonella* isolates. b; Overall prevalence of HMRGs and Biocide resistance among 143 *Salmonella* isolates. *zntB* confers resistance to zinc (Zn); *pcoABCDERS* confer resistance to copper (Cu); *silAB* confers resistance to silver (Ag); *terDW* confers resistance to tellurium (Te). Biocide resistance genes *qacE* and *qacL* confer resistance to quaternary ammonium compounds.

Although *Salmonella* is an intestinal infection, it can effectively survive outside its host through the formation of biofilms, which serve as a crucial survival adaptation mechanism. The phenotypic biofilm formation potential of 143 *Salmonella* strains at 37°C is shown in (Fig. 4a). Overall, more than 90% of isolates were biofilm producers, exhibiting varied levels of production. Specifically, 69.93% of isolates had weak biofilm production, 13.98% exhibited moderate production, and only 8.93% have been identified as high biofilm producers. Conversely, 7.69% of isolates were non-biofilm producers. Thirteen heavy metal resistance genes (HMRGs) of *arsC, merA, pcoS pcoA, pcoB, pcoE pcoC, pcoD, pcoR, silA, silB, terD,* and *terW*, as well as two biocides resistant genes *qacL* and *qacE*, were identified in *Salmonella* isolates (Fig. 4b). Overall, *pcoS* was the most common (27.3%; 39/143) gene, while *arsC* had the lowest frequency (3.5%; 5/143).

### Detection of plasmid replicons

**Fig. 5:**
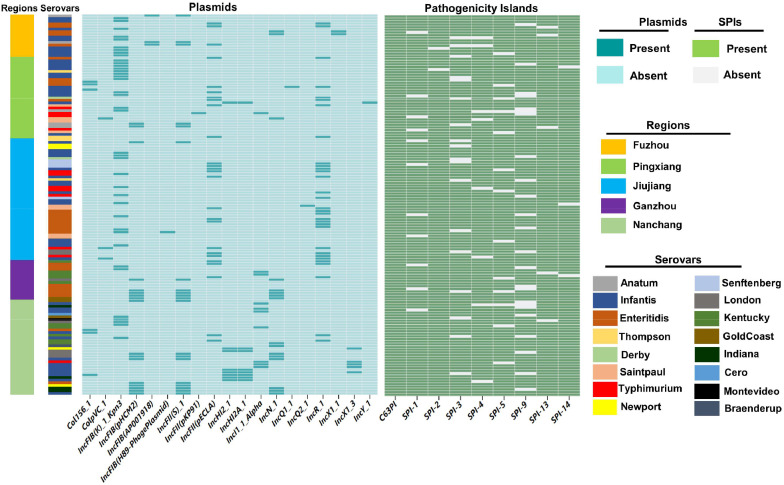
Distribution of plasmids replicons and pathogenicity Islands (PIs) among 143 *Salmonella* isolates.

Plasmid replicons were widely distributed among the isolates. A total of 19 distinct plasmid replicons were identified among 143 *Salmonella* genomes (Fig. 5). In total, *IncFIB(K)* (27.27%; 39/143) was the most prevalent plasmid replicon, followed by *IncR* (20.98%; 30/143) and *IncFII* (*pECLA*) (17.48%; 25/143).

### Detection of Pathogenic Island (PIs) and virulence factors

**Fig. 6:**
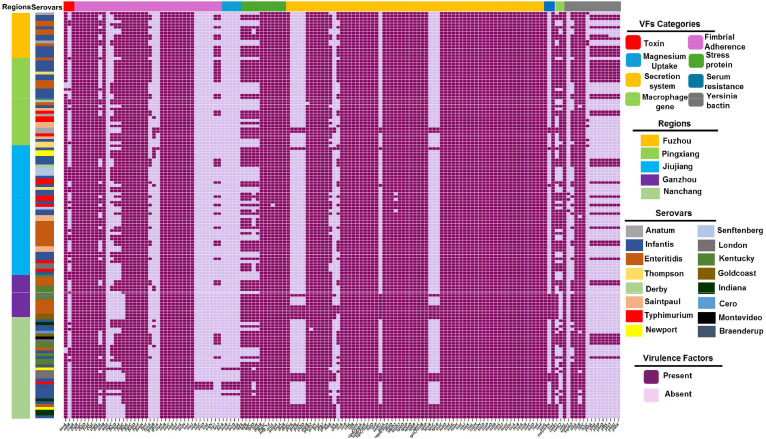
Distribution of virulence genes among the 143 studied *Salmonella* isolates.

Genetic virulence variables were analyzed in 143 genomes to further understand the pathogenicity repertoire of *Salmonella* isolates. *Salmonella* genomes exhibit a common pathogenic island (PI), C63PI, present in 100% of genomes followed by SPI-2 (98.6%), SPI-14 (97.9%), SPI-13 (96.5%) and SPI-4 (95.1%) (Fig. 5). The complete virulence gene profile of each isolate of *Salmonella* is displayed in (Fig. 6). A total of 145 virulence genes associated with nine classes, including *Salmonella* invasion, adhesion, colonization, toxicity, secretion, serum resistance, iron and magnesium intake, and survival, were classified. All isolates exhibited a predominant presence of virulence genes, primarily associated with the type III secretion system (T3SS), encoded by *Salmonella* pathogenicity islands SPI-2 and SPI-1. A variety of additional gene clusters, including those associated with antibiotic resistance, iron and magnesium uptake, fimbrial adherence, flagellar apparatus biosynthesis determinants, and colonization, were conserved. On the other hand, unique virulence genes (*iucABCD* and *pefABCD*) were also detected at different rates (Fig. 6). Some of the genes that encode the virulent yersinia bactin operon (*fyuA, irp12, ybt*), the iron uptake (*entE*), the cytolethal distending toxin Gene (*cdtB*) and the Typhoidal toxin were also found in few isolates.

## Discussion

*Salmonella* infections, a prevalent foodborne disease, currently present a significant global public health concern. Importantly, Raw chicken meat is a significant source of *Salmonella* for humans, with estimates suggesting that approximately 30% of global foodborne Salmonellosis cases may be associated with poultry meat (Hoffmann, et al., 2017). Human infection mainly spreads through the consumption of contaminated raw poultry products or *Salmonella* colonized chicken (Siddique, et al., 2024b). Bird flocks in poultry farms may acquire *Salmonella* infections by vertical or horizontal transmission (Liljebjelke, et al., 2005). In our study, we scrutinized the antibiotic resistance, and epidemiological and genomic features of *Salmonella* isolated from poultry faeces in five representative cities of Jiangxi Province, China, by using WGS data together with precise bioinformatics methods.

This study indicates that the overall prevalence of *Salmonella*, identified using conventional microbiological techniques, was approximately 7.94% in samples collected from poultry farms in Jiangxi Province. Nanchang had the highest prevalence of *Salmonella* (12.0%), followed by Pingxiang (10.33%) and Jiujiang (7.5%). These findings were consistent with a previous study in Sichuan Province, China (Li, Lai, Wang, Liu, Li, Liu, Shen and Wu, 2013), which showed a prevalence (165/1382, 11.9%) of *Salmonella* in ducks and chicken farms. However, studies conducted in Ethiopia, the USA, and Ecuador indicated a lower prevalence of *Salmonella*, determined through conventional microbiological methods, ranging from 4.7% to 6.5% (Alali, et al., 2010; Eguale, 2018; Mejía, et al., 2020). In particular, the existence of *Salmonella* in animal hosts without symptoms is a major threat to public health. Enforcing essential hygiene protocols in farms and live markets effectively prevents the transmission of *Salmonella* to humans, primarily via the food chain (McMillan, et al., 2020; Saharan, et al., 2020).

Epidemiological surveillance of *Salmonella* serovars needs to be given high priority throughout the food chain to ensure consumer safety. According to serovar prediction, *S*. Infantis (ST32) and *S*. Enteritidis (ST11) were the most commonly detected in this study. These results were consistent with a previous study in which *S*. Infantis and *S*. Enteritidis were the predominant serovars isolated from chicken farms in Jiangsu Province, China (Meng, et al., 2025) and *S*. Enteritidis was the most common serovars Japanese poultry broilers (Yokoyama, et al., 2019). According to a previous study, *S*. Infantis was the predominant serovar in Jordan isolated from poultry companies (Gharaibeh, et al., 2024). The detection rate of *S*. Enteritidis in China was 1.5% from 2006 to 2012 and increased to 15.1% from 2013 to 2021 (Yue, et al., 2022). Recent reports indicate a rise in *S*. Infantis infections in both poultry and humans across various countries (Dionisi, et al., 2011; Montone, et al., 2023; Nógrády, et al., 2008). In the United States, human infections with *S*. Infantis increased by 167% from 2001 to 2016 (Mattock, et al., 2024). There are significant variations in *Salmonella* serovars across countries, including within various regions of the same country (Zhou, 2023). A prior study demonstrated that *S*. Reading was the predominant serovar isolated from turkeys in North America (Miller, et al., 2020). The differences in the prevalence of various *Salmonella* serotypes across studies may be attributed to factors such as sample locations, timing of sampling, and the hygienic practices implemented on the farms being examined.

Antibiotics have long been used as a common therapy for bacterial infections, including *Salmonella*, in China. The prolonged and widespread application of antibiotics in veterinary medicine has led to the emergence of antimicrobial resistance in animals and livestock (Aslam, et al., 2021). In this study, about 60.84% of the *Salmonella* isolates were multidrug-resistant (MDR). The resistance rate of *Salmonella* to multidrug resistance (MDR) was lower than previously recorded in commercial layer flocks in Türkiye (Çokal, et al., 2020). Furthermore, *Salmonella* strains isolated from broilers exhibited higher levels of multidrug resistance compared to those obtained from laying hens (İnce and Akan, 2023). The increasing prevalence of *Salmonella* strains exhibiting resistance to essential antibiotics poses a significant threat to One Health. The first-line antibiotics, amoxicillin/clavulanic acid, tetracycline, and streptomycin, used to treat pathogenic infections in livestock globally, have high levels of resistance (88.8%, 82.5%, and 49.7% respectively). These outcomes align with those of Liu et al. (Liu, et al., 2021), who found that *Salmonella* isolates from farm animals had a higher rate of resistance among these antibiotics. In this study, quinolone and beta-lactam resistance were also present in a considerable amount of the *Salmonella* isolates, which is consistent with earlier research that indicated significant resistance to these antibiotics in *Salmonella* isolates attained from farm chicken. These outcomes prompt a major concern for public health because these antibiotic classes are essential for the treatment of human infections with salmonellosis.

Our analysis reveals a comprehensive understanding of the mechanisms contributing to the phenotypic resistance of the isolates examined, as we successfully identified multiple ARGs using WGS analysis. This study identified 61 resistance genes that confer resistance to seven different classes of antibiotics. The most frequently found ARGs were *aph(*3′*)-Ia, qnrS1, aph(*3′'*)-Ib*, and *tetA*. These findings were consistent with previous findings (Chen, et al., 2019; Laughlin, et al., 2019; Zhou, et al., 2018). The majority of PMQR genes are present in animals from China (Jacoby, et al., 2015), with the exception of one isolate, YC300, which contains the *qnrE1* gene. The *qnrE1* gene is predominantly located in South America (Monte, et al., 2019). A total of 24 beta-lactam resistant genes were also found in this study. Our research confirmed the results that indicated beta-lactam genes may be spreading throughout China, particularly in chicken meat samples (Wang, et al., 2020). A previous report showed that *bla_TEM_* was the most prevalent (88.8%) (Gharaibeh, Lafi, Allah and Al Qudsi, 2024) ARG in the *Salmonella* isolates. The significant level of ESBL and quinolone resistance identified in this study is alarming for public health. High coherence was found in the relationship between phenotypic and genotypic resistance, particularly for aminoglycosides, trimethoprim-sulfamethoxazole, and tetracycline. Our findings agreed with those of earlier studies on isolates of *Salmonella* (Galán-Relaño, et al., 2022). A limited number of isolates exhibited false negative results, specifically those that were phenotypically resistant yet genotypically susceptible. The observed phenomenon is likely due to the existence of novel ARG variants that remain undiscovered since they are not incorporated into the reference database used for prediction (Pornsukarom, van Vliet and Thakur, 2018). Consequently, the phenotypic test continues to be the gold standard for assessing how bacteria behave in response to antibiotics (White, et al., 2001). An in-depth study of AMR in *Salmonella* from poultry may clarify the current patterns of drug resistance in *Salmonella* and help in the development of effective prevention and control strategies to address the rising problem of drug resistance in *Salmonella*.

This study examined the biofilm production capability at 37°C for 24 hrs to investigate the impact of environmental factors in chicken farms on *Salmonella* biofilm formation. We found that over 90% of strains had the ability to produce biofilms, demonstrating a range of potential from weak to strong. Our research findings on *Salmonella* align with previous studies of *Salmonella enterica* serovars since all studied strains demonstrated biofilm production (Borges, et al., 2018; Merino, et al., 2019).

Animal feed additives that contain metals are also frequently employed. Metals, such as zinc, copper, manganese, chromium, and cobalt are often used in animal feed due to their growth-stimulating and antimicrobial properties (Schwan, et al., 2023). The existence of residual heavy metal ions in poultry production has recently become unknown (Aljohani, 2023). Heavy metal contamination in meat and the presence of these pollutants in the environment pose risks to food safety and human health (Mustafa, et al., 2021). In this study, thirteen heavy metal resistance genes (HMRGs) *arsC, merA, pcoS, pcoA, pcoB, pcoE, pcoC, pcoD, pcoR, silA, silB, terD,* and *terW*, as well as two biocides resistant genes *qacL* and *qacE*, were identified in *Salmonella* isolates. These HMRGs confer resistance to various heavy metals, including silver, copper, cobalt, mercury, and ternium, as well as two biocides-resistant genes, *qacL* and *qacE*. Antibiotic resistance and heavy metal resistance may be related, as indicated by the coexistence of HMRGs and ARGs (Di Cesare, et al., 2016; Li, et al., 2022a) . As previously reported, the *arsB* gene had a strong association with *sul2* and *int1*, while the beta-lactam resistance genes in Enterobacteriaceae were linked to metal resistance genes, *pcoA, merA, silC*, and *arsA* (Yang, et al., 2020). The coexistence of heavy metals and antibiotics on the same mobile genetic elements, such as plasmids, leads to an interaction between heavy metal resistance genes and antibiotic resistance genes, often resulting in co-resistance to both. Sublethal amounts of heavy metals can increase the amount of ARG and aid in the development of bacterial resistance to antibiotics (Poole, 2017).

Plasmids are known for significantly contributing to the spread of ARGs, HMRGs, virulence genes, and other traits that provide fitness advantages. The results of our investigation identified nineteen distinct plasmids in the examined *Salmonella* isolates, with *IncX1* and *IncFll(S)* predominating. Moreover, our findings indicate that these plasmids were present in numerous serotypes and regions, suggesting potential widespread distribution among different origins. These plasmids are also related to resistance to different types of antibiotic classes, such as sulfonamides, beta-lactams, aminoglycosides, and tetracyclines. These results were consistent with previous findings in which the *IncX1* plasmid was dominant in the *Salmonella* strains isolated from food animals (Zhang, et al., 2018). This is particularly concerning as multiple studies have identified IncX1 and other conjugative plasmids in clinical isolates of pathogenic *Salmonella* (Cao, et al., 2023; Garrido, et al., 2024).

Virulence genes in *Salmonella* play a crucial role in their fitness, including persistence in specific niches and effective pathogenesis. They contribute to pathogenicity and host colonization by facilitating attachment, invasion, and replication within host cells, while evading host defenses through mechanisms such as adhesion systems, capsules, flagella, and toxins. This study revealed that all *Salmonella* isolates possessed multiple fimbrial genes (*fim, inv*, and *csg*) and secretion systems that contribute to cell invasion and bacterial survival within phagocytes. According to a previous finding, fimbriae play an important role in mediating adhesion between the bacterium and host cells (Adedokun, et al., 2023). The cytolethal distending toxin (CDT), also known as typhoid toxin, was initially believed to be limited to the serovars Typhi and Paratyphi A. It induces cell arrest as a result of DNA damage, which contributes to the manifestation of typhoid fever in humans (Fierer, 2022). In our study, the CDT genes of *cdtB*, which encode typhoid toxin production, were found in a few *Salmonella* isolates. This finding is in agreement with prior studies that related the *cdtB* gene to isolates of food animals (Elbediwi, et al., 2020). Importantly, the *cdtB* gene was identified in non-typhoidal *Salmonella* isolates causing invasive infections in humans (McMillan, Jackson and Frye, 2020). Additionally, the *spV* and *pef* loci are crucial to the virulence mechanisms of NTS strains (Xu, et al., 2020b). Previous studies indicate that the presence of this gene cluster typically leads to enhanced growth at the infection site (Wang, Li, Liu, Cheng and Su, 2020), biofilm formation, and an increase in overall pathogenicity in chickens (Khan, et al., 2019). The existence of *Salmonella* with these virulence factors isolated from food animals and their products poses a significant hazard to public health, with the potential for serious illness in end consumers.

The main limitation of this study is the insufficient number of tested chicken samples, which restricts generalized conclusions regarding the prevalence of *Salmonella*. Consequently, future research should incorporate a larger sample size. Another limitation is the absence of information concerning drug use in the poultry industry. The lack of such information prohibits us from properly assessing the influence of small changes in the level of use of various antibiotics on resistance in bacteria obtained from chickens. As a result, interdisciplinary efforts are required to reduce the use of some antibiotics that have demonstrated significant resistance patterns in chicken production. New measures should be taken immediately to limit the use of some of the highly resistant drugs in animals, ultimately helping to minimize the selection forces that produce antimicrobial resistance.

## Conclusion

In conclusion, this study demonstrates a diverse range of AMR *Salmonella* serovars isolated from poultry farms across various regions, highlighting the necessity for stringent surveillance of antimicrobial resistance in food production animals in Jiangxi Province. The complete resistome profile identified in this study will help to design AMR control programs to reduce the load of ARGs in the food chain. This study revealed an adequate concordance between phenotypic antimicrobial susceptibility and predicted genotypes derived from WGS results, indicating that this technology may be utilized alongside phenotypic tests for surveillance purposes. Information from this study serves as a valuable reference for future research and can guide public health authorities in efforts to limit the spread of ARGs, thereby contributing to the global fight against the AMR threat.

## Availability of data

The raw nucleotide sequence reads were uploaded to the National Centre for Biotechnology Information's (NCBI) Short Read Archive (SRA) database using BioProject accession number PRJNA994834 (Supplementary Table S2).

## Declaration of competing interest

None.

## Appendix Supplementary materials

Image, application 1

## Footnotes

Supplementary material associated with this article can be found, in the online version, at doi:10.1016/j.psj.2025.105197.
